# Effect of direct oral anticoagulant therapy on pulmonary artery clot dissolution in intermediate high-risk pulmonary thromboembolism

**DOI:** 10.1186/s12959-024-00631-6

**Published:** 2024-07-10

**Authors:** Hiroya Hayashi, Akihiro Tsuji, Akiyuki Kotoku, Hiroyuki Endo, Naruhiro Nishi, Takatoyo Kiko, Ryotaro Asano, Jin Ueda, Tatsuo Aoki, Tetsuya Fukuda, Takeshi Ogo

**Affiliations:** 1 Division of Pulmonary Circulation, Department of Cardiovascular Medicine, National Cerebral and Cardiovascular Center, 6-1, Kishibe-Shimmachi, Suita, Osaka 564-8565 Japan; 2 Department of Radiology, National Cerebral and Cardiovascular Center, Suita, Osaka Japan

**Keywords:** Intermediate high-risk pulmonary thromboembolism, Direct oral anticoagulants, Computed tomography obstruction index, Warfarin, Right ventricular/left ventricular ratio

## Abstract

**Background:**

Direct oral anticoagulants are the established drugs for treating pulmonary thromboembolism. The advantage of direct oral anticoagulants over conventional therapy for clot lysis and right ventricular unloading in the acute phase remains unclear. This study aimed to evaluate the effect of acute treatment with direct oral anticoagulants on clot dissolution and right ventricular unloading in intermediate high-risk pulmonary thromboembolism.

**Methods:**

Thirty patients with intermediate high-risk pulmonary thromboembolism admitted between November 2012 and December 2018 were included; 21 and 9 were treated with direct oral anticoagulants and conventional therapy, respectively. We compared the efficacy of clot dissolution and right ventricular unloading for intermediate high-risk pulmonary thromboembolism between direct oral anticoagulant and conventional therapy in the acute phase. Efficacy was assessed by computed tomography obstruction index, right/left ventricular ratio, and brain natriuretic peptide levels between baseline and at discharge.

**Results:**

Computed tomography obstruction index, right ventricular/left ventricular ratio, and brain natriuretic peptide levels were significantly lower at discharge than at admission in both groups. The rate of improvement in computed tomography obstruction index was significantly higher in the direct oral anticoagulant therapy group than in the conventional therapy group (64 ± 15% vs. 47 ± 16%; *p* = 0.01). There were no significant differences in the rate of improvement in right ventricular/ left ventricular ratio and brain natriuretic peptide levels between the two groups.

**Conclusions:**

Compared with conventional therapy, direct oral anticoagulants significantly reduced pulmonary artery clot burden conventional therapy in the acute treatment of intermediate high-risk pulmonary thromboembolism.

## Background

Pulmonary thromboembolism (PTE) is a common disease with an estimated annual incidence of 70 cases per 100,000 population [1]. Intermediate high-risk PTE with right ventricular (RV) dysfunction and positive cardiac laboratory biomarkers is associated with a poor prognosis alongside high-risk PTE [2, 3]. Prompt thrombus lysis and stabilization of RV heart unloading are essential for patients with intermediate high-risk PTE since the severity can easily be aggravated [4].

Newer mainstream direct oral anticoagulants (DOACs) that inhibit factor Xa or thrombin have a faster onset of action than warfarin [5–10]. Early thrombus lysis is essential to achieve good prognosis in patients with intermediate high-risk PTE. Although DOACs are useful for treating intermediate high-risk PTE in clinical practice, few studies have attempted to evaluate the extent to which treatment with DOACs dissolves pulmonary artery clots in the acute phase of intermediate high-risk PTE [2, 11].

The thrombus clot burden in PTE can be quantified using various computed tomography (CT) imaging techniques, such as the CT obstruction index [12, 13]. The CT obstruction index can be obtained by calculating the number of clots in the large pulmonary arteries [12, 13]. Some studies have suggested that clot burden scoring could be used to predict short-term mortality in patients with acute PTE [14, 15].

This study aimed to evaluate the effect of acute treatment with DOACs, including the degree of thrombotic dissolution, using CT imaging and brain natriuretic peptide (BNP) levels in intermediate high-risk PTE.

## Methods

### Study population

**Fig. 1:**
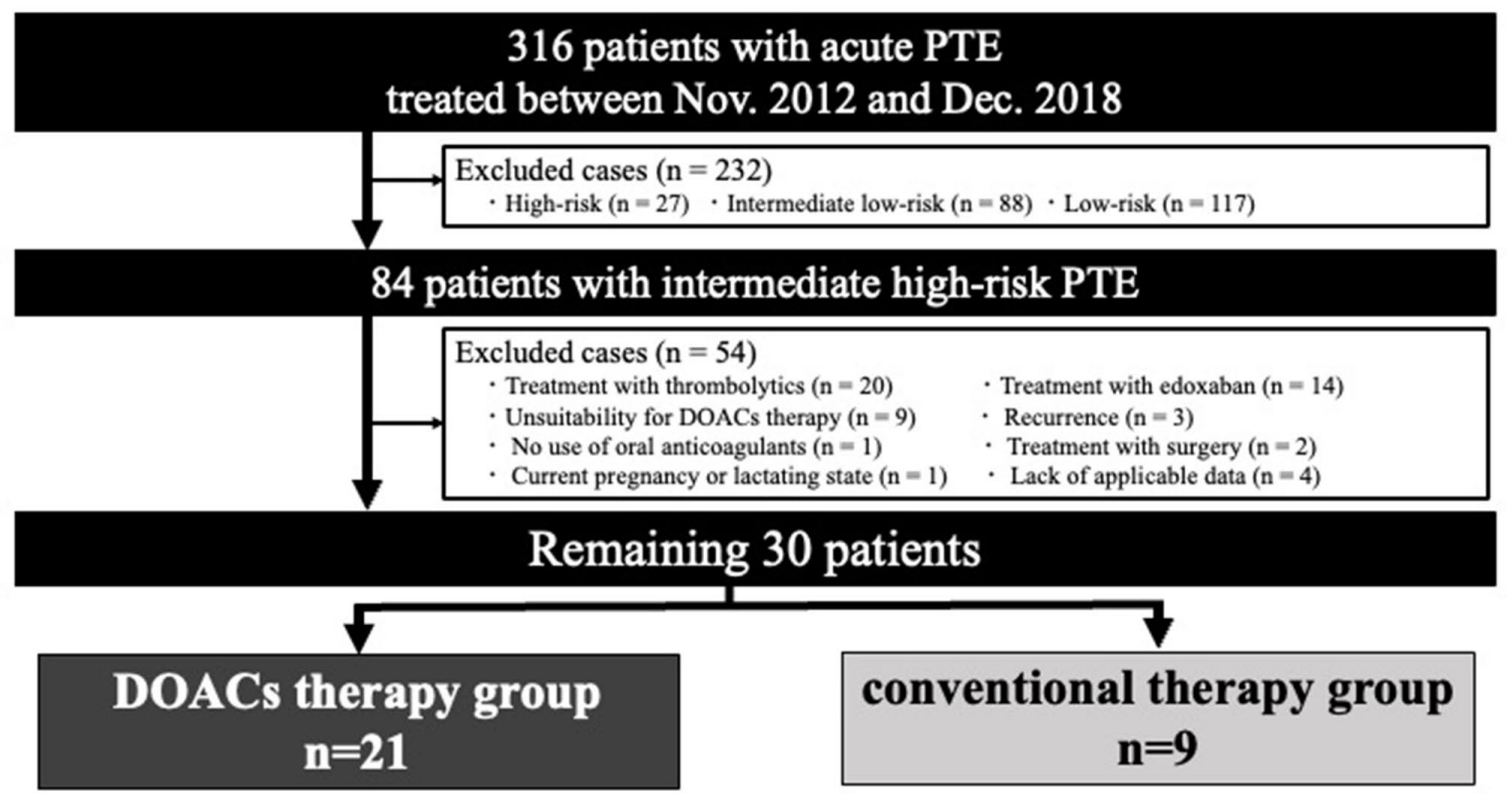
Study flowchart. This retrospective observational study included 84 patients with intermediate high-risk PTE treated between November 2012 and December 2018. Thirty patients were enrolled in the study. DOACs: direct oral anticoagulants; PTE: pulmonary thromboembolism

This retrospective, nonrandomized, single-center study initially screened the data of 316 patients treated with acute PTE at our center between November 2012 and December 2018. We compared the efficacy of DOACs and conventional therapy with warfarin following parental anticoagulation therapy for 84 patients with intermediate high-risk PTE. We selected rivaroxaban or apixaban as DOAC therapy, which eliminated the need for continuous parenteral therapy and undergoing intensive periods in the initial phase. Edoxaban was excluded from this study because it is not indicated intensive therapy. Patients in the DOAC therapy group were treated with DOACs 2 h after the administration of unfractionated heparin (UFH) bolus 5000 U. This is because the time-to-peak efficacy for DOACs is approximately 2 h [16], but recurrent PTE may be fatal even within 2 h in intermediate high-risk cases [17]. Rivaroxaban was started at 15 mg twice daily for the first 3 weeks, followed by 15 mg once daily, which was the dosage approved in Japan. Apixaban was also started at 10 mg twice daily for 1 week, followed by 5 mg twice daily. Patients in the conventional therapy group were treated with parenteral anticoagulation therapy, followed by warfarin. We aimed for an activated partial thromboplastin time that was 1.5–2.5 times that of the baseline as the therapeutic range of UFH and prothrombin time as an international normalized ratio of 1.5–2.5 as the therapeutic range of warfarin according to the Japanese guidelines [18]. Subsequently, we compared the treatment effectiveness in the acute phase of DOAC therapy versus that of conventional therapy. The exclusion criteria were as follows: treatment with thrombolytics (*n* = 20), treatment with edoxaban (*n* = 14), unsuitability for DOAC therapy (continuous infusion of UFH, inappropriate DOAC dose, or change in DOACs) (*n* = 9), recurrence (*n* = 3), no use of oral anticoagulants (*n* = 1), treatment with surgery (*n* = 2), current pregnancy or lactating status (*n* = 1), and lack of applicable data (*n* = 4). Finally, 30 patients were included in this study (Fig. 1).

### Study methods

Intermediate high-risk PTE was defined as positivity for cardiac laboratory biomarkers (cardiac troponin T and/or plasma BNP), signs of RV dysfunction on an imaging test, and pulmonary embolism severity index (PESI) Class III–V or simplified PESI ≥ 1 [3]. RV dysfunction was defined as an RV/left ventricular (LV) ratio ≥ 0.9 on contrast-enhanced CT [19]. Ventricular diameters were measured by identifying the maximal distance between the ventricular endocardium and interventricular septum perpendicular to the long axis of the heart [20]. According to previous reports, the CT obstruction index was defined as follows: the arterial tree of the lung was regarded as having 18 segmental arteries (3 on the right upper lobe, 2 on the left upper lobe, 2 each in the middle lobe and lingula, 5 in the right lower lobe, and 4 in the left lower lobe) and scored according to the number of pulmonary arterial branches with thrombi [21]. These measurements were confirmed by two certified cardiologists (Readers 1 and 2) to evaluate interobserver reproducibility. To assess intra-observer reproducibility, Reader 1 repeated the same procedure twice, with an interval of at least 1 month. Treatment effectiveness in the acute phase was evaluated based on changes in the CT obstruction index, RV/LV ratio on contrast-enhanced CT, and plasma BNP levels at admission versus discharge. The improvement rate was defined as the difference between values at admission and discharge divided by values at admission and multiplied by 100.

### Acquisition of CT

All patients were examined in the supine position using a single-source 320-detector-row CT scanner (Aquilion ONE; Canon Medical Systems, Tochigi, Japan) or a dual-source CT scanner (Somatom Definition Flash, Siemens Healthcare, Forchheim, Germany).

CT pulmonary angiography (CTPA) was performed using Aquilion ONE. The default kilovoltage (kVp) setting for the CTPA protocol was 120 kVp. The quality reference milliampere (mA) value was 300. Dual-energy CT (DECT) was performed using Somatom Definition Flash. The kVp settings for the DECT protocol were 100 kVp and Sn 140 kVp. The quality reference mA value for tube A was 100.

Patients received an injection of a non-ionic iodine contrast medium containing 370 mg iodine/mL (Oypalomin 370, Fuji Pharma corporation, Tokyo, Japan) through a 20-gauge cannula inserted in the forearm antecubital vein. This contrast medium was administered as a fractional dose at 17.3 mg iodine/kg of body weight/s for 20 s. This was followed by administering a 40:60 mixture of contrast medium and saline for 10 s. A bolus-tracking technique was applied, and imaging was automatically initiated 5 s after the enhancement level reached a threshold of 150 Hounsfield units in the region of interest placed in the main pulmonary trunk. CTPA or DECT images were obtained with a slice thickness of 1 mm and an increment of 1 mm.

Overall, 21 (70%) and all 30 patients underwent CT at our facility before and after treatment, respectively. Before treatment, 11 patients (52%) underwent CTPA using the Aquilion ONE scanner. After treatment, 29 patients (97%) underwent DECT using the Somatom Definition Flash scanner.

### Statement of ethics

This study was approved by the National Cerebral and Cardiovascular Center Research Ethics Committee (R19020-3), which waived the requirement for informed consent according to the Japanese Clinical Research Guidelines because of the study’s retrospective observational nature. A public announcement of the study was rather made upon request from the Ethics Committee and Guidelines.

### Statistical analysis

Categorical variables are presented as numbers and percentages. Continuous variables are presented as means and standard deviations or medians and interquartile ranges based on their distributions. Categorical variables were compared using the chi-square test when appropriate; otherwise, the Fisher’s exact test was used. Continuous variables were compared using Student’s t-test or Wilcoxon’s rank-sum test based on their distributions. A two-way random, single-measure (absolute agreement) intraclass correlation coefficient (ICC) was used to assess the differences between the features initially generated by Readers 1 and 2 and between the twice-generated features by Reader 1. An ICC value < 0.40 indicated poor reliability, values between 0.41 and 0.59 indicated fair reliability, values between 0.60 and 0.74 indicated good reliability, and values between 0.75 and 1.00 indicated excellent reliability. This descriptive statistic can be used when quantitative measurements are performed on units organized into groups. It describes how strongly units in the same group are similar and has been previously reported to be a reliable method for evaluating data reproducibility [22, 23]. In this study, all tests were two-tailed, and statistical significance was set at *p* < 0.05. These analyses were performed using the JMP software (version 13.0.0; SAS, Cary, NC, USA) or SPSS software (29.0.2.0; SPSS, Inc., Chicago, IL, USA).

## Results

The clinical characteristics of the study population are summarized in Table 1. Of the 30 included patients, 21 (70%) were treated with DOACs and 9 (30%) with conventional therapy. In the DOAC therapy group, 11 patients were treated with apixaban and 10 with rivaroxaban. At baseline, patients treated with DOACs were younger (69.2 ± 13.5 vs. 82.0 ± 11.3 years, *p* = 0.019) and had a higher mean hemoglobin level (13.7 ± 1.2 vs. 11.9 ± 1.8 g/dL, *p* = 0.004) than that observed among those treated with conventional therapy. The proportion of inferior vena cava filter was higher in the conventional therapy group than in the DOAC therapy groups but was not associated with bleeding risk or clinical course. There were no significant intergroup differences in sex, body mass index, mean hospitalization duration, vital signs, PESI score, BNP level, CT obstruction index, or RV/LV ratio at baseline.

**Table 1:** Patient baseline characteristics by study group

Characteristic	DOAC therapy (*n* = 21)	Conventional therapy (*n* = 9)	*P* value
Mean age (years)	69.2 ± 13.5	82.0 ± 11.3	0.019
Male sex, N (%)	6 (28.6)	2 (22.3)	0.72
BMI (kg/m^2^)	25.2 ± 3.9	23.8 ± 6.2	0.44
Hospitalization duration (days)	19.1 ± 12.4	21.4 ± 9.0	0.61
Systolic BP (mmHg)	138 ± 28	139 ± 21	0.91
Diastolic BP (mmHg)	84 ± 14	77 ± 16	0.23
Heart rate (bpm)	96 ± 15	92 ± 16	0.59
PaO_2_/FiO_2_ ratio	257 ± 93	316 ± 190	0.27
PESI score	103 ± 22	112 ± 25	0.20
Simple PESI score	1.5 ± 0.7	1.9 ± 0.8	0.43
Hemoglobin (g/dL)	13.7 ± 1.2	11.9 ± 1.8	0.004
Creatinine (mg/dL)	0.83 ± 0.28	0.74 ± 0.16	0.40
CCr (mL/min)	73.7 ± 29.4	55.6 ± 19.2	0.10
d-dimer (µg/mL)	21.6 ± 18.6	27.5 ± 40.9	0.59
Troponin T (ng/mL)	0.096 ± 0.09	0.100 ± 0.11	0.92
BNP (pg/mL)	258 (127–353)	227 (107–272)	0.48
TRPG (mmHg)	41.3 ± 22.1	46.0 ± 9.2	0.54
TAPSE (mm)	17.3 ± 5.7	15.4 ± 5.9	0.42
CT obstruction index	59.0 ± 14.4	59.6 ± 10.2	0.91
RV/LV ratio on CT	1.39 ± 0.33	1.42 ± 0.24	0.82
IVC filter, N (%)	2 (10)	5 (56)	0.006
Antiplatelet therapy, N (%)	0 (0)	1 (9)	0.30
DVT, proximal, N (%)	7 (33)	5 (56)	0.26
DVT, distal, N (%)	11 (52)	3 (33)	0.34
**Risk factor**			
Cancer, N (%)	2 (10)	1 (11)	0.89
Surgery, N (%)	0 (0)	3 (33)	0.005
Rest, N (%)	2 (10)	2 (22.2)	0.35
Thrombophilia, N (%)	2 (10)	0 (0)	0.34
Obesity, N (%)	9 (43)	3 (33)	0.63
Dehydration, N (%)	1 (5)	0 (0)	0.51
Major trauma, N (%)	0 (0)	1 (11.1)	0.12
Paralytic stroke, N (%)	0 (0)	0 (0)	1.00
Drug, N (%)	1 (5)	0 (0)	0.51
IBD, N (%)	0 (0)	0 (0)	1.00
Unprovoked, N (%)	12 (57)	5 (56)	0.94

Values are expressed as the median and interquartile range (IQR) or mean ± SD

Values in parentheses are percentages

DOACs: direct oral anticoagulants; BMI: body mass index; BP: blood pressure; PaO_2_/FiO_2_: ratio of arterial oxygen partial pressure to fraction inspired oxygen; PESI: pulmonary embolism severity index; CCr: creatinine clearance; BNP: brain natriuretic peptide; TRPG: tricuspid regurgitant pressure gradient; TAPSE: tricuspid annular plane systolic excursion; CT: computed tomography; RV: right ventricular; LV: left ventricular; IVC: inferior vena cava; DVT: deep vein thrombosis; IBD: inflammatory bowel disease

The average duration of UFH use in the conventional treatment group was 6.9 ± 2.3 days, with an average dose of 18,000 ± 3,000 units per day. The mean final activated partial thromboplastin time (APTT) value was 77.7 ± 15.7 s, and all patients achieved the target APTT value within 24 h. There were no significant intergroup differences in most risk factors; however, only the rate of surgical treatment was lower rate in the DOAC therapy group than in the conventional therapy group (0 vs. 33%, *p* = 0.005). We evaluated the thrombophilia test results for all patients at PTE diagnosis. Two patients in the DOAC therapy group experienced thrombophilia; one had protein S deficiency, and the other had antiphospholipid syndrome. We re-performed the thrombophilia tests after more than 3 months. One patient with protein S deficiency in the DOAC group had lower protein S activity and antigen level, even in the chronic phase (after more than 3 months). The other patient with antiphospholipid syndrome had positive anti-cardiolipin IgG results, which were not affected by DOACs in the acute and chronic phases. The CT obstruction index at discharge was significantly lower in the DOAC therapy group than in the conventional therapy group (22.4 ± 10.3 vs. 30.6 ± 7.9%, *p* = 0.042); however, there were no significant differences were observed between the two groups in terms of the RV/LV ratio and BNP levels at discharge, despite comparable follow-up periods (0.86 ± 0.12 vs. 0.98 ± 0.20, *p* = 0.051; 21 [14–43] vs. 33 [19–61] pg/mL, *p* = 0.35) (Table 2).

**Table 2:** Patient discharge characteristics by study group

Characteristic	DOAC therapy (*n* = 21)	Conventional therapy (*n* = 9)	*P* value
Days to follow-up contrast-enhanced CT (days)	11.6 ± 5.9	10.2 ± 2.6	0.50
CT obstruction index	22.4 ± 10.3	30.6 ± 7.9	0.042
RV/LV ratio	0.86 ± 0.12	0.98 ± 0.20	0.051
Days to follow-up laboratory data (days)	14.4 ± 12.3	13.2 ± 4.6	0.78
BNP (pg/mL)	21 (14–43)	33 (19–61)	0.35

Values are expressed as median and interquartile range (IQR) or mean ± SD

DOACs: direct oral anticoagulants; CT: computed tomography; RV: right ventricular; LV: left ventricular; BNP: brain natriuretic peptide

**Fig. 2:**
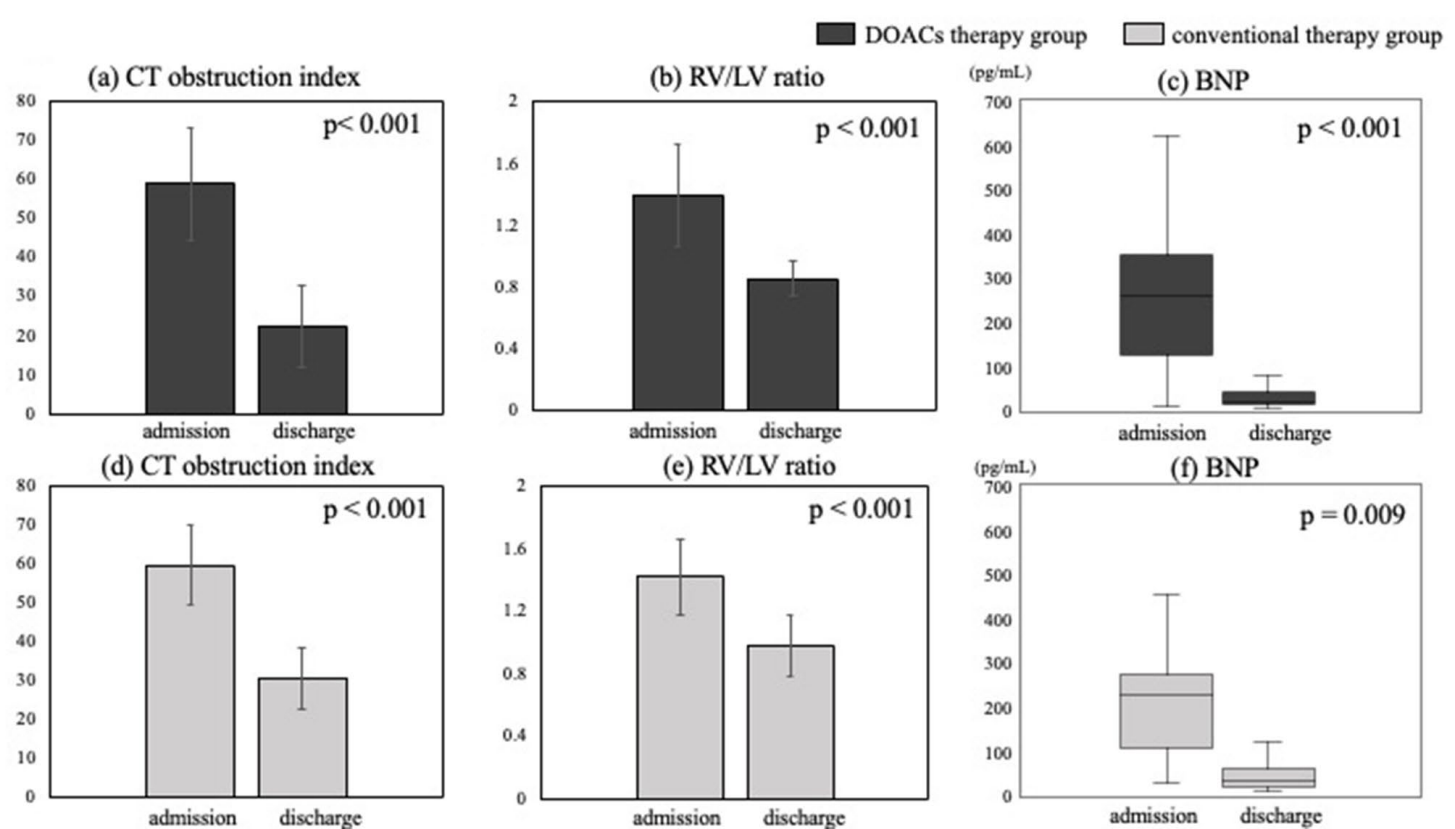
Changes in CT obstruction index, RV/LV ratio, and BNP levels between admission and discharge. (**a**) The CT obstruction index was significantly lower at discharge than at admission in the DOAC therapy group. (**b**) The RV/LV ratio was significantly lower at discharge than at admission in the DOAC therapy group. (**c**) BNP levels were significantly lower at discharge than at admission in the DOAC therapy group. (**d**) The CT obstruction index was significantly lower at discharge than at admission in the conventional therapy group. (**e**) The RV/LV ratio was significantly lower at discharge than at admission in the conventional therapy group. (**f**) BNP levels were significantly lower at discharge than at admission in the conventional therapy group. BNP: brain natriuretic peptide; CT: computed tomography; DOACs: direct oral anticoagulants; LV: left ventricular; RV: right ventricular

**Fig. 3:**
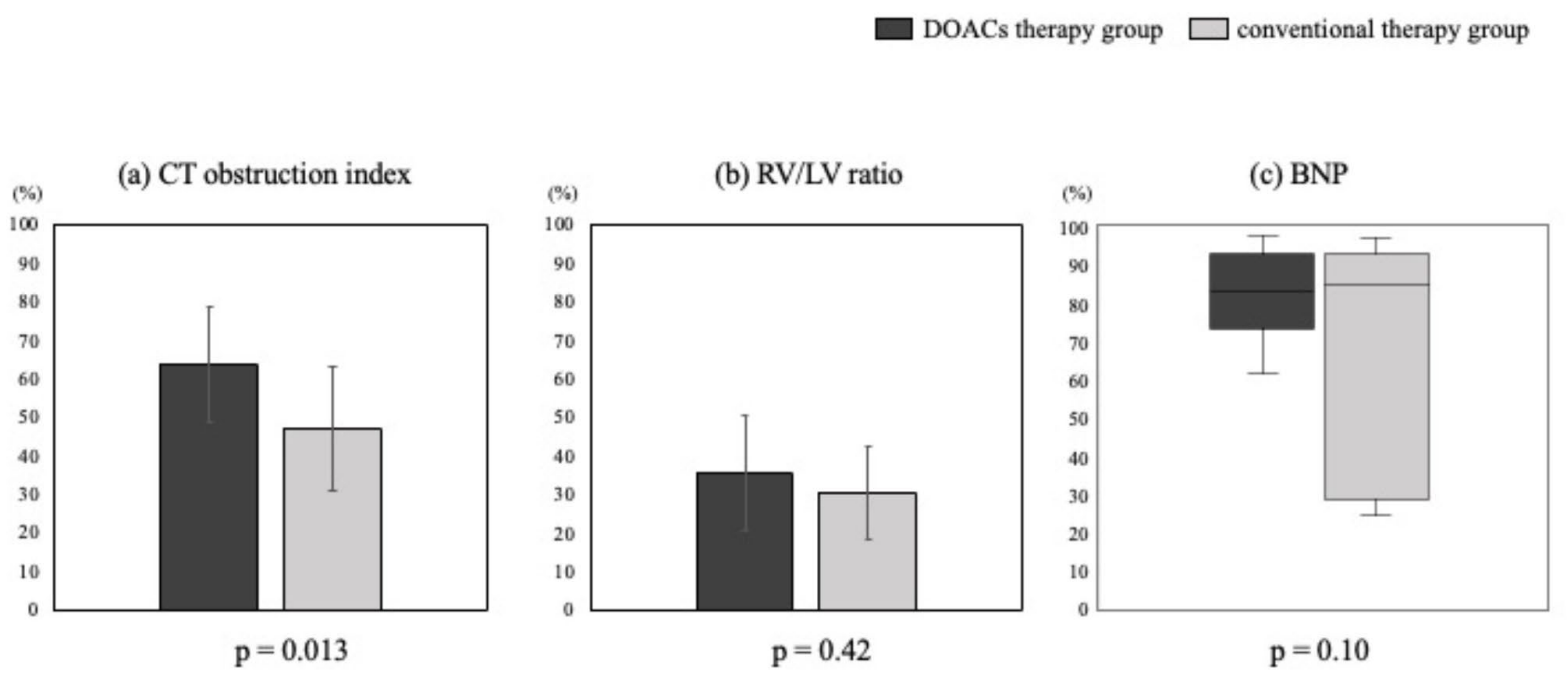
Improvement rate in CT obstruction index, RV/LV ratio, and BNP levels between admission and discharge. The improvement ratio of the CT obstruction index in the DOAC therapy group was 64%, while that in patients in the conventional therapy group was 47%. The improvement ratio of the RV/LV ratio in the DOAC therapy group was 36%, whereas that in the conventional therapy group was 31%. The improvement rate of BNP levels in the DOAC therapy group was 83%, whereas that in the conventional therapy group was 85%. BNP: brain natriuretic peptide; CT: computed tomography; DOACs: direct oral anticoagulants; LV: left ventricular; RV: right ventricular

The DOAC therapy group showed significant improvement in the CT obstruction index, RV/LV ratio, and BNP level at discharge compared with those at admission (59.0 ± 14.4 vs. 22.4 ± 10.3, *p* < 0.001; 1.39 ± 0.33 vs. 0.86 ± 0.12, *p* < 0.001; 258 [127–353] vs. 21 [14–43] pg/mL, *p* < 0.001, respectively) (Fig. 2a, b, and c). Similarly, the conventional therapy group showed significant improvement at discharge compared with that at admission (59.6 ± 10.2 vs. 30.6 ± 7.9, *p* < 0.001; 1.42 ± 0.24 vs. 0.98 ± 0.20, *p* < 0.001; 227 [107–272] vs. 33 [19–61] pg/mL, *p* = 0.009, respectively) (Fig. 2d, e, and f). Figure 3 shows the improvement rate of the CT obstruction index, RV/LV ratio, and BNP levels. The improvement rate of the CT obstruction index in the DOAC therapy group was 64 ± 15%, while that in the conventional therapy group was 47 ± 16% (Fig. 3a). The improvement rate of the CT obstruction index was significantly higher in the DOAC therapy group (*p* = 0.013). The improvement rate of the RV/LV ratio in the DOAC therapy group was 35 ± 16%, while that in the conventional therapy group was 31 ± 12% (Fig. 3b). There was also no significant intergroup difference in the improvement rate of the RV/LV ratio (*p* = 0.42). The improvement rate of BNP levels in the DOAC therapy group was 83% (73–93%), while that in the conventional therapy group was 85% (28–93%) (Fig. 3c). There was no significant intergroup difference in the improvement rate of BNP levels (*p* = 0.10).

Satisfactory inter- and intraobserver reproducibility of CT imaging was achieved. The reproducibility of the improvement in the CT obstruction index was good between the two readers (ICC range: 0.85 [0.71–0.93]) or between the reader’s first and second-extracted features (ICC range: 0.95 [0.89–0.98]). The reproducibility of the improvement in RV/LV ratio was good between the two readers (ICC range: 0.92 [0.81–0.96]) or between the reader’s first and second-extracted features (ICC range: 0.94 [0.87–0.97]). These results suggest that the CT obstruction index and RV/LV ratio are highly reproducible.

No thrombotic recurrence was observed during acute treatment in either group. No major bleeding episode occurred in both groups during this study period. None of the patients in either group experienced worsening of disease severity. All the patients were discharged alive.

## Discussion

This study showed that in intermediate high-risk PTE, DOACs were more effective than conventional therapy for pulmonary artery clot dissolution assessed using the CT obstruction index in the acute phase. In contrast, no significant differences in the DOACs were shown for RV unloading assessed by the RV/LV ratio and BNP levels.

The DOAC therapy group had a significantly higher improvement rate in CT obstruction index than that had by the conventional therapy group. These findings may suggest that DOACs are more effective in preventing exacerbations in patients with intermediate high-risk PTE. This is supported by the notion that the extent of thrombolysis during the acute phase of intermediate high-risk PTE is a prognostic factor for patient outcomes [14]. DOACs have more rapid and stable anticoagulant effects than that had by conventional therapy. Moreover, we selected DOACs with intensive periods, resulting in powerful anticoagulant effects in the acute phase. These DOAC effects may lead to significant differences in thrombus reduction between both groups. In contrast, in the conventional therapy group, the initial continuous parenteral therapy was limited to UFH, which might be regarded as less optimal than low molecular weight heparin [24]. In Japan, low molecular weight heparin has not been approved for venous thromboembolism (VTE). Moreover, we aimed for a prothrombin time as an international normalized ratio of 1.5–2.5 as the therapeutic range of warfarin, which was lower than that recommended worldwide. These factors may also lead to less anticoagulant effects in the conventional therapy group.

However, there were no significant differences in the improvement rate of RV/LV ratio and BNP level between the two groups. The RV/LV ratio is an indicator of RV dysfunction in patients with PTE [25]. The abrupt increase in RV pressure and volume leads to an increase in RV wall tension and stretch [3]. However, the effect of pulmonary artery thrombotic dissolution is followed by the improvement of RV wall tension and stretch. Hence, it might take a little more time to achieve the improvement of the RV/LV ratio, leading to no significant difference between the two groups in this study. BNP is a prognostic marker for patients with PTE [26]. BNP is released in response to myocardial stretch. It only takes a few hours for BNP levels to increase significantly after the onset of acute myocardial stretch [26]. Thus, both groups had rapid and dramatic improvements in BNP levels, resulting in no significant difference.

Cancer-associated thrombosis (CAT) can cause recurrent VTE and bleeding. CAT includes arterial thrombosis, VTE, and nonbacterial thrombotic endocarditis, and the increase in VTE in patients with cancer has become a particular problem [27–29]. In contrast, several recent studies have demonstrated that DOACs are effective against CAT [30–32]. Three patients with cancer (two treated with DOAC therapy and one with conventional therapy) were included in this study, and neither thrombotic recurrence nor major bleeding occurred during this study period.

This study has several limitations. First, because this was a single-center study, the number of patients was small. Although few baseline characteristics were statistically different, some numerical differences in these may have existed and might have become significant with increased sample size. Second, this was an observational retrospective study; thus, selection bias was inevitable. Third, this study only evaluated the acute phase effects, and the chronic phase effects were not determined. Fourth, the treatment strategies for VTE could have varied widely during the study period, which could have influenced the current results. Finally, only a few patients with cancer were included in this study. Patients with cancer are prone to thrombosis owing to the cancer or its treatment and are also prone to bleeding. Therefore, considering these differences between patients with and without cancer is essential.

## Conclusions

This study suggests that DOAC therapy may reduce pulmonary artery thrombotic volume better than that observed with conventional therapy in patients with intermediate high-risk PTE in the acute phase. In contrast, no significant differences were found in the DOAC therapy group for RV unloading. Further investigation through prospective studies is necessary.

## Data Availability

No datasets were generated or analysed during the current study.

## Abbreviations

APTT: activated partial thromboplastin time
BNP: brain natriuretic peptide
CAT: cancer-associated thrombosis
CT: computed tomography
CTPA: CT pulmonary angiography
DECT: dual-energy CT
DOACs: direct oral anticoagulants
ICC: intraclass correlation coefficient
LV: left ventricular
PESI: pulmonary embolism severity index
PTE: pulmonary thromboembolism
RV: right ventricular
UFH: unfractionated heparin
VTE: venous thromboembolism
